# Association Between Fragmentation of Care and Delivery of Adjuvant Chemotherapy in Patients Traveling to High-Volume Hospitals for Pancreatic Adenocarcinoma

**DOI:** 10.1245/s10434-025-18539-4

**Published:** 2025-10-16

**Authors:** Alexa J. Hughes, Kristen N. Kaiser, Emma Holler, Brian M. Ruedinger, Anita A. Turk, Cary Jo R. Schlick, Michael G. House, Karl Y. Bilimoria, Ryan J. Ellis

**Affiliations:** 1 Surgical Outcomes and Quality Improvement Center (SOQIC), Department of Surgery, Indiana School of Medicine, Indianapolis, IN USA; 2 Division of Surgical Oncology, Department of Surgery, Indiana University School of Medicine, Indianapolis, IN USA; 3 Division of Hematology/Oncology, Department of Medicine, Indiana University School of Medicine, Indianapolis, IN USA

**Keywords:** Pancreatic ductal adenocarcinoma, Adjuvant chemotherapy, Care fragmentation, Travel distance, High-volume hospitals, Health care access

## Abstract

**Background:**

Surgical care for pancreatic ductal adenocarcinoma (PDAC) is increasingly centralized to high-volume hospitals (HVHs), prompting many patients to travel farther for resection. While surgery is centralized, adjuvant chemotherapy is often delivered locally, resulting in care fragmentation. The implications of this separation on chemotherapy receipt and survival are unclear. This study evaluated associations between travel distance, care fragmentation, and receipt of adjuvant chemotherapy in patients undergoing upfront PDAC resection at HVHs and assessed how these factors influenced overall survival.

**Methods:**

Patients with non-metastatic PDAC who underwent upfront resection at HVHs (≥20 pancreatectomies/year) were identified from the National Cancer Database (2007–2021). The cohort was stratified by adjuvant chemotherapy receipt, travel distance (deciles D1–D10), and care fragmentation. Multivariable logistic regression assessed factors associated with chemotherapy receipt; Cox proportional hazards models evaluated survival.

**Results:**

Among 17,807 patients treated at 97 HVHs, 10,200 (57%) received adjuvant chemotherapy. Patients traveling ≥14 miles (≥D4) were less likely to receive adjuvant chemotherapy (D4 odds ratio [OR] 0.85; 95% confidence interval [CI] 0.73–0.99; *P*=0.04). Patients experiencing care fragmentation were more likely to receive adjuvant therapy (64.3% vs. 54.4%, OR 1.51; 95% CI 1.35–1.69; *P*<0.001). Travel ≥20 miles (≥D5) was associated with higher mortality (hazards ratio [HR] 1.12; 95% CI 1.02–1.23; *P*=0.01). Conversely, receipt of adjuvant chemotherapy (HR 0.77; 95% CI 0.73–0.81; *P*<0.001) and fragmented care (HR 0.89; 95% CI 0.84–0.93; *P*<0.001) were associated with improved survival.

**Conclusions:**

Longer travel distance was associated with lower chemotherapy receipt and worse survival. Care fragmentation was linked to improved treatment access and survival, underscoring the need for coordinated cross-institutional care.

**Supplementary Information:**

The online version contains supplementary material available at 10.1245/s10434-025-18539-4.

Pancreatic ductal adenocarcinoma (PDAC) is an aggressive malignancy and remains the fourth leading cause of cancer-related death in the United States, with a 5-year overall survival rate around 10%.^1,2^ National Comprehensive Cancer Network guidelines recommend that resectable PDAC should be managed with a combination of surgical resection and chemotherapy.^3–6^ Despite these recommendations, receipt of guideline-based, multimodal treatment is highly variable, ranging from 35 to 79%.^7–12^ This variability is influenced by multiple factors, including postoperative complications, early disease progression, care coordination challenges, and healthcare disparities such as patient access to surgical care.^8,13^

Centralization of specialized oncologic surgery such as pancreatectomy to high-volume hospitals (HVHs) is increasingly recommended to optimize outcomes. HVHs have demonstrated lower perioperative morbidity and better survival than lower-volume centers.^14–17^ Patients who undergo surgery at HVHs experience fewer postoperative complications and better short-term recovery, which enhances receipt of adjuvant therapy, an essential component for improving long-term survival in patients with PDAC.^8^ However, the limited number of HVHs in the United States can impose substantial travel burdens on patients. This travel burden can result in patients receiving adjuvant therapy at facilities closer to home, leading to care fragmentation.^18,19^ While dispersing care across multiple facilities can improve access to highly specialized treatment, the literature is mixed on whether fragmentation of care improves or worsens clinical outcomes.^20–23^ Difficulties in characterizing the intersection between fragmentation, care coordination, and quality of oncologic care are due to nuanced decisions regarding delivery of multimodality therapy as well as variable treatment sequencing.

To address this, the authors identified delivery of adjuvant chemotherapy after upfront pancreatectomy for PDAC as a test case to assess the interplay between care fragmentation and adjuvant care delivery after upfront pancreatectomy at an HVH. This test case was chosen because pancreatectomy is increasingly centralized to HVHs and essentially all patients are recommended to receive adjuvant therapy after upfront pancreatic resection, limiting guideline variability within the treatment pathway.^14,24,25^ The objectives of this study were to (1) evaluate associations between travel distance, care fragmentation, and receipt of adjuvant chemotherapy in patients undergoing upfront resection for PDAC at HVHs and (2) assess how travel distance and care fragmentation are associated with receipt of adjuvant chemotherapy and survival in this population.

## Methods

### Data Source

The National Cancer Database (NCDB) is a joint initiative of the American College of Surgeons Commission on Cancer (CoC) and the American Cancer Society.^26^ Data are collected from CoC-accredited facilities in the United States, capturing approximately 70% of new cancer diagnoses in the United States. The NCDB contains patient demographic information with cancer diagnosis, pathology, treatment, and outcomes. Data entry in the NCDB is protocolized and entered by a trained cancer registrar that utilizes a standard data dictionary.^27^ All data in the NCDB are deidentified. This study was reviewed by the Indiana University IRB office and determined to be non-human-subjects research. The study followed the Strengthening the Reporting of Observational Studies in Epidemiology (STROBE) Reporting guidelines.^28^

### Patient Selection

The NCDB was retrospectively reviewed from 2007 to 2021 on adult patients diagnosed with PDAC who underwent an upfront pancreatic resection. Patients diagnosed with PDAC from 2007 to 2021 were included to maximize sample size and capture long-term trends in travel distance, care fragmentation, and adjuvant chemotherapy use. The cohort start year coincided with the publication of CONKO-001, which established adjuvant chemotherapy as standard of care for resected pancreatic cancer. Annual pancreatectomy volume was defined as the total number of pancreatectomies done at each unique facility divided by the total number of years with at least one pancreatectomy in the NCDB. HVH was defined as 20 pancreatectomies a year based on Leapfrog’s Standards.^29^ Adjuvant chemotherapy was defined as chemotherapy receipt within 12 weeks following definitive surgical resection. Patients were excluded if they received neoadjuvant therapy, had metastatic disease, had a previous malignancy, had surgery at a low-volume facility, or had missing survival data.

Distance traveled for each patient was estimated between the residential zip code and the address of the treating hospital using Haversine’s formula (crowfly distance in miles). Patients with a crowfly distance of >250 miles were excluded as we considered them a clinically unique subset of patients that encompassed healthcare tourism, which is beyond the scope of this study.^30^

Care structure was classified as fragmented or coordinated care. Fragmentation of care was defined as receiving a component of their cancer care (surgery or chemotherapy) at more than one facility based on more than one reporting facility in the NCDB.^20,31^ Patients who had one reporting facility were assumed to receive their surgery and chemotherapy at the same facility and subsequently defined as coordinated care.^32^

### Statistical Analysis

The relationship between distance traveled and mortality was examined using locally weighted scatterplot smoothing (LOWESS) statistical modeling because of the nonlinear relationship between distance traveled and overall survival (Supplemental Figure S1). LOWESS modeling is a non-parametric regression model used to analyze the relationship between variables without assuming a specific functional form. Therefore, travel distance was stratified into deciles to analyze mortality risk more accurately by comparing trends within distinct distance groups rather than assuming a uniform linear effect.

Demographic, socioeconomic, clinical, and histopathologic factors of patients receiving adjuvant therapy were compared to patients who did not receive adjuvant chemotherapy. Baseline demographics across travel distance groups were calculated and compared using the χ2 test for categorical variables and either a t-test or the Mann–Whitney U test for continuous variables as appropriate. Multivariable logistic regression was performed to identify factors associated with the receipt of adjuvant chemotherapy, adjusting for relevant demographic and clinical characteristics, including, age, sex, race, insurance status, income level, Charlson-Deyo comorbidity score, clinical stage, location of tumor, and year of diagnosis. Multiple interaction terms were tested, including care fragmentation and distance, which were not significant and therefore no interaction terms were utilized or stratified within the final model. Multivariable Cox proportional hazards modeling was utilized to adjust for confounding and preserve sample size in survival analysis. All adjusted models incorporated covariates designated a priori. Two-way interactions between care structure and other variables, including age, year of diagnosis, clinical stage, and travel distance, were tested within the Cox model. Non-significant interaction terms (*P*>0.05) were dropped from the final model. Missing data were handled using complete case analysis. All statistical analyses were conducted using Stata (College Station, TX, USA) and R version 4.1.1 for Mac (Vienna, Austria), with a significance threshold of *P*<0.05.

## Results

### Patient Cohort Characteristics

Over 15 years, 17,807 patients with PDAC who underwent upfront pancreatic resection at 97 HVHs met inclusion criteria. Most patients were male (52%), were aged >60 years (77%), were white (80%), and had a Charleson-Deyo score of 0 (63%). Patients traveled an average of 44 (± 49) miles to their treatment facility. Overall, 10,200 patients (57%) received adjuvant chemotherapy, and 5,166 patients (29%) experienced fragmented care. The majority of patients had tumors in the head of the pancreas (72%) and stage II disease (77%). Additional demographic and treatment characteristics for the study population are summarized in Table 1.

**Table 1:** Demographics of overall cohort

Parameter	No. (%)
Total	17,807
Distance	
Average crowfly (miles)	44 ± 49
Sex	
Male	9,253 (52)
Female	8,554 (48)
Age group (years)	
<50	1,090 (6.1)
50–59	3,241 (18)
60–69	6,163 (35)
70–79	5,439 (31)
≥80	1,874 (11)
Race and ethnicity	
Non-Hispanic white	14,186 (80)
Non-Hispanic Black	1,547 (8.7)
Hispanic	8,15 (4.6)
Asian	535 (3.0)
Other/unknown	724 (4.1)
Income quartiles	
Lowest (<$46,227)	2,515 (14)
2^nd^	3,545 (20)
3^rd^	4,188 (24)
Highest (>$74,063)	7,346 (42)
Insurance status	
Private	6,593 (37)
Uninsured+ Medicaid	1,120 (6.3)
Medicare	9,646 (54)
Other/unknown	448 (2.5)
Charlson-Deyo score	
0	11,261 (63)
1	4,666 (26)
2	1,225 (6.9)
3	655 (3.7)
Pathological stage	
I	2,531 (14)
II	13,747 (77)
III	1,529 (8.6)
Tumor location	
Head	12,740 (72)
Body	1,322 (7.4)
Tail	2,074 (12)
Other/overlapping	1,671 (9.4)
Year treated	
2007	1,031 (5.8)
2008	1,093 (6.1)
2009	1,198 (6.7)
2010	1,282 (7.2)
2011	1,302 (7.3)
2012	1,382 (7.8)
2013	1,353 (7.6)
2014	1,425 (8.0)
2015	1,464 (8.2)
2016	1,294 (7.3)
2017	1,215 (6.8)
2018	1,097 (6.2)
2019	1,015 (5.7)
2020	844 (4.7)
2021	812 (4.6)
Adjuvant chemotherapy	
Received	10,200 (57)
Fragmented care	5,166 (29)
Coordinated care	12,641 (71)

### Factors Associated with Receipt of Adjuvant Chemotherapy

Patients were significantly less likely to receive adjuvant chemotherapy if they traveled more than 14 miles to an HVH for surgery. Compared with those in the nearest decile (D1), patients in the fourth decile (D4, mean 16 ± 2 miles) had reduced odds of receiving adjuvant chemotherapy (D4 63.4%, odds ratio [OR] 0.85; 95% confidence interval [CI] 0.73–0.99; *P*=0.04).The likelihood of receiving adjuvant chemotherapy progressively decreased with greater travel distance, with patients in the farthest decile (D10) having the lowest odds (OR 0.40; 95% CI 0.32–0.50; *P*<0.001) (Table 2). Patients also had decreased odds of receiving adjuvant chemotherapy if they were Black (56.2%; OR 0.83; 95% CI 0.74–0.94; *P*=0.004), had Medicaid or no insurance (55.6%;OR 0.67; 95% CI 0.58–0.78; *P*<0.001) or Medicare (51.8%; OR 0.85; 95% CI 0.78–0.93; *P*<0.001), or if they were aged ≥60 years (60–69 years, 61.7%; OR 0.68; 95% CI 0.58–0.80; *P*<0.001; 70–79 years, 52.3%; OR 0.47; 95% CI 0.39–0.56; *P*<0.001; ≥80 years, 34%; OR 0.2; 95% CI 0.17–0.25; *P*<0.001).

**Table 2:** Factors associated with receipt of adjuvant chemotherapy in overall cohort

Parameter	Rate (%)	OR (95% CI)	*P*-value
Distance deciles (Average crowfly, miles)			
D1 (3 ± 1)	62.7	ref	ref
D2 (7 ± 1)	63.7	1.00 (0.87–1.14)	0.99
D3 (11 ± 1)	64.2	0.96 (0.83–1.12)	0.62
D4 (16 ± 2)	63.4	0.85 (0.73–0.99)	0.04
D5 (22 ± 2)	61.5	0.78 (0.66–0.91)	0.002
D6 (30 ± 3)	56.7	0.65 (0.55–0.77)	<0.001
D7 (42 ± 4)	55.8	0.65 (0.56–0.76)	<0.001
D8 (60 ± 7)	53.7	0.61 (0.52–0.73)	<0.001
D9 (91 ± 12)	47.5	0.50 (0.41–0.61)	<0.001
D10 (162 ± 36)	43.4	0.40 (0.32–0.50)	<0.001
Fragmented care			
No	54.4	ref	ref
Yes	64.3	1.51 (1.35–1.69)	<0.001
Sex			
Male	58.1	ref	ref
Female	56.3	0.97 (0.91–1.04)	0.44
Age group (years)			
<50	69.6	ref	ref
50–59	66.6	0.82 (0.69–0.97)	0.02
60–69	61.7	0.68 (0.58–0.80)	<0.001
70–79	52.3	0.47 (0.39–0.56)	<0.001
≥80	34.0	0.20 (0.17–0.25)	<0.001
Race and ethnicity			
NH-white	57.6	ref	ref
NH-Black	56.2	0.83 (0.74–0.94)	0.004
Hispanic	58.3	0.87 (0.71 –1.07)	0.19
Asian	60.0	0.87 (0.71–1.05)	0.14
Other/unknown	50.1	0.74 (0.56–0.97)	0.03
Income quartiles			
Lowest	50.2	ref	ref
2^nd^	52.7	1.08 (0.96–1.21)	0.18
3^rd^	57.1	1.18 (1.04–1.33)	0.007
Highest	62.1	1.32 (1.12–1.56)	0.001
Insurance status			
Private	65.7	ref	ref
Uninsured + Medicaid	55.6	0.67 (0.58–0.78)	<0.001
Medicare	51.8	0.85 (0.78–0.93)	<0.001
Other/unknown	56.0	0.84 (0.67–1.05)	0.122
Charlson-Deyo Score			
0	58.6	ref	ref
1	56.4	0.97 (0.89–1.06)	0.51
2	53.6	0.85 (0.75–0.97)	0.01
3	47.3	0.64 (0.53–0.77)	<0.001
Pathological stage			
I	45.0	ref	ref
II	59.1	1.91 (1.71–2.12)	<0.001
III	61.3	1.67 (1.40–2.00)	<0.001
Tumor location			
Head	58.0	ref	ref
Body	55.9	1.02 (0.89–1.16)	0.80
Tail	58.6	1.08 (0.95–1.23)	0.25
Other/overlapping	51.2	0.82 (0.73–0.92)	0.001
Year treated			
2007	46.3	ref	ref
2008	50.9	1.18 (0.95–1.47)	0.13
2009	52.3	1.25 (1.02–1.54)	0.03
2010	55.4	1.45 (1.11–1.89)	0.007
2011	55.8	1.42 (1.12–1.80)	0.004
2012	56.9	1.45 (1.13–1.87)	0.003
2013	57.1	1.53 (1.23–1.90)	<0.001
2014	59.5	1.70 (1.36–2.14)	<0.001
2015	62.9	1.90 (1.47–2.46)	<0.001
2016	59.2	1.70 (1.32–2.20)	<0.001
2017	61.2	1.86 (1.42–2.43)	<0.001
2018	60.7	2.11 (1.59–2.79)	<0.001
2019	59.6	2.12 (1.63–2.77)	<0.001
2020	57.7	1.93 (1.43–2.61)	<0.001
2021	62.7	2.42 (1.89–3.11)	<0.001

CI, confidence interval; OR, odds ratio

**Fig. 1:**
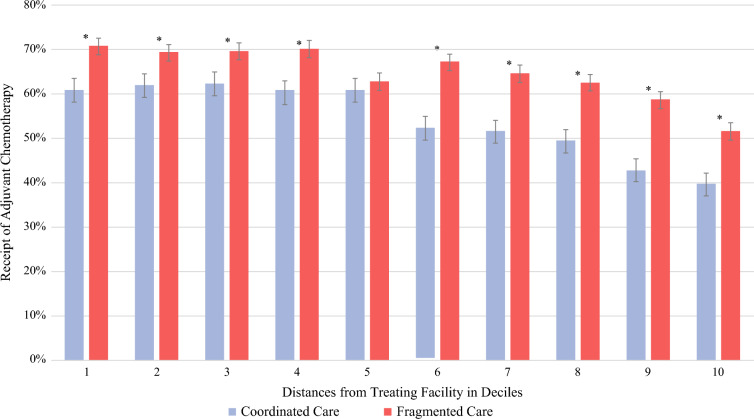
Patient travel distance and care fragmentation influence on receipt of adjuvant chemotherapy. *Denotes significance with *P*-value <0.05

Patients were more likely to receive adjuvant chemotherapy if they had more advanced disease (61.3% for stage III vs 45.0% for stage I; OR 1.67; 95% CI 1.40–2.0; *P*<0.001) or if they received fragmented care (64.3% vs 54.4% with coordinated care; OR 1.51; 95% CI 1.35–1.69; *P*<0.001). Across all travel distance deciles, patients receiving fragmented care had higher rates of adjuvant chemotherapy than those with coordinated care (Fig. 1). Pairwise comparisons within each decile demonstrated that these differences were statistically significant in 9 of the 10 deciles (*P*<0.001, Fig. 1). The most pronounced differences were observed at or above the 6^th^ decile, where chemotherapy receipt declined sharply for patients with coordinated care but remained consistently high among those with fragmented care (Fig. 1). Among patients who received coordinated care, adjuvant chemotherapy rates declined steadily with increasing travel distance, from 61.0% in the closest decile (D1) to 40.0% in the farthest decile (D10). Rates of adjuvant therapy also increased over time, with the highest odds of receiving chemotherapy in 2021 (62.7%; OR 2.42; 95% CI 1.89–3.11;* P*<0.001), relative to the reference year 2007.

### Survival Analysis

When analyzing overall survival, patients had improved survival if they were female (HR 0.96; 95% CI 0.93–1.00; *P*=0.041). Compared with patients aged <50 years, those aged 70–79 years had a 24% increased risk of death (HR 1.24; 95% CI 1.12–1.38; *P*<0.001), and those aged ≥80 years had a 44% increased risk (HR 1.44; 95% CI 1.30–1.59; *P*<0.001). Those with Medicaid or who were uninsured (HR 1.10; 95% CI 1.01–1.19; *P*=0.02) and those on Medicare (HR 1.10; 95% CI 1.03–1.17; *P*=0.006) had worse survival than patients with private insurance. Compared with patients treated in 2007, those treated in later years had progressively lower HRs, with the lowest observed in 2018 (HR 0.63; 95% CI 0.56–0.71; *P*<0.001), 2019 (HR 0.65; 95% CI 0.57–0.75; *P*<0.001), and 2020 (HR 0.65; 95% CI 0.56–0.76; *P*<0.001) (Table 3).

**Table 3:** Multivariable cox proportional hazards model for overall mortality

Parameter	HR (95% CI)	*P*-value
Sex		
Male	ref	
Female	0.96 (0.93–1.00)	0.041
Age group (years)		
<50	ref	
50–59	1.06 (0.97–1.16)	0.20
60–69	1.07 (0.98–1.17)	0.13
70–79	1.24 (1.12–1.38)	<0.001
≥80	1.44 (1.30–1.59)	<0.001
Race and ethnicity		
Non-Hispanic white	ref	
Non-Hispanic Black	1.02 (0.95–1.09)	0.64
Hispanic	0.82 (0.70–0.96)	0.012
Asian	0.81 (0.71–0.92)	0.001
Other/unknown	1.00 (0.89–1.13)	0.98
Income quartiles		
Lowest	ref	
2^nd^	1.01 (0.93–1.09)	0.85
3^rd^	0.95 (0.89–1.02)	0.19
Highest	0.89 (0.83–0.96)	0.001
Insurance status		
Private	ref	
Uninsured + Medicaid	1.10 (1.01–1.19)	0.020
Medicare	1.10 (1.03–1.17)	0.006
Other/unknown	1.12 (0.96–1.30)	0.15
Charlson–Deyo score		
0	ref	
1	1.04 (1.00–1.08)	0.076
2	1.15 (1.06–1.25)	0.001
3	1.43 (1.29–1.58)	<0.001
Pathological stage		
I	ref	
II	2.49 (2.29–2.70)	<0.001
III	3.52 (3.15–3.94)	<0.001
Tumor location		
Head	ref	
Body	0.96 (0.90–1.02)	0.18
Tail	0.90 (0.86–0.95)	<0.001
Other/overlapping	0.92 (0.86–0.99)	0.023
Year treated		
2007	ref	
2008	1.00 (0.91–1.09)	0.92
2009	0.98 (0.89–1.07)	0.59
2010	0.92 (0.84–1.01)	0.087
2011	0.87 (0.80–0.95)	0.001
2012	0.83 (0.75–0.91)	<0.001
2013	0.85 (0.77–0.94)	0.001
2014	0.82 (0.74–0.91)	<0.001
2015	0.77 (0.69–0.86)	<0.001
2016	0.72 (0.65–0.80)	<0.001
2017	0.67 (0.60–0.75)	<0.001
2018	0.63 (0.56–0.71)	<0.001
2019	0.65 (0.57–0.75)	<0.001
2020	0.65 (0.56–0.76)	<0.001
Distance (deciles)		
D1	ref	
D2	1.06 (0.97–1.16)	0.17
D3	1.05 (0.97–1.13)	0.24
D4	1.03 (0.92–1.15)	0.63
D5	1.12 (1.02–1.23)	0.014
D6	1.17 (1.07–1.29)	0.001
D7	1.19 (1.09–1.29)	<0.001
D8	1.22 (1.11–1.34)	<0.001
D9	1.12 (1.01–1.24)	0.035
D10	1.13 (1.01–1.26)	0.028
Fragmented care		
No	ref	
Yes	0.89 (0.84–0.93)	<0.001
Adjuvant chemotherapy		
No	ref	
yes	0.77 (0.73–0.81)	<0.001

CI, confidence interval; HR, hazard ratio

**Fig. 2:**
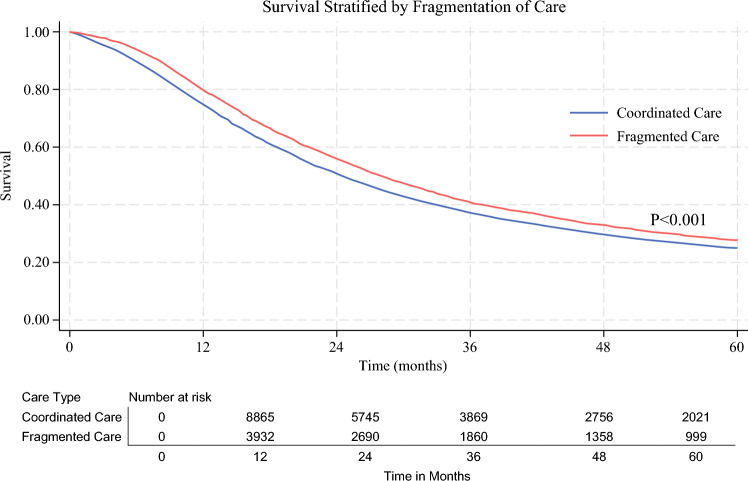
Kaplan–Meier analysis stratified by fragmented care

Greater travel distance was associated with decreased survival. Compared with those in the nearest decile, patients in the 6th through 10th distance deciles had significantly increased mortality risk, with HRs ranging from 1.12 to 1.22 (all *P*<0.05). Conversely, fragmented care was associated with improved survival (HR 0.89; 95% CI 0.84–0.93; *P*<0.001) (Fig. 2). Overall, receipt of adjuvant chemotherapy was associated with improved survival (HR 0.77; 95% CI 0.73–0.81; *P*<0.001).

## Discussion

This study examined the relationship between travel distance, care fragmentation, and the receipt of adjuvant chemotherapy among patients with PDAC who underwent upfront resection at HVHs. The results demonstrate that patients who traveled farther were significantly less likely to receive adjuvant chemotherapy. In contrast, those who traveled shorter distances and those who experienced fragmented care had higher rates of adjuvant therapy and improved overall survival. Importantly, the survival advantage observed among patients receiving fragmented care appears to be driven by improved access to adjuvant chemotherapy, underscoring the value of systemic therapy in long-term outcomes. This study highlights that care fragmentation, in this specific context, was associated with improved delivery of adjuvant chemotherapy and better survival outcomes. These findings illustrate the challenges posed by care coordination, travel distance, and treatment access in centralized healthcare models.

The centralization of complex oncologic surgery has improved perioperative outcomes but may inadvertently impede equitable access to care. In the context of pancreatic cancer, there has been a movement toward centralization of surgical procedures, especially pancreatic head resections, driven by evidence that HVHs offer superior perioperative outcomes.^33,34^ While some countries enforce centralization through policy and surgical case minimums, the United States relies on voluntary adherence to recommended surgical quality metrics. Organizations such as Leapfrog and the American Cancer Society recommend that pancreatectomies be performed at institutions completing at least 20 procedures annually.^29^ As a result, patients often travel substantial distances to receive surgical care at HVHs.^33,34^ While centralization to HVHs improves perioperative outcomes, it can hinder delivery of follow-up multimodal therapy due to travel burden. Patients in this study who traveled farther were less likely to receive adjuvant therapy and experienced worse overall survival, highlighting potential consequences of travel burden. As surgical care continues to centralize, developing robust inter-institutional pathways and communication systems will be essential to support equitable delivery of postoperative therapy. This challenge is particularly relevant in PDAC, where timely adjuvant chemotherapy is crucial for improving survival following upfront resection.^11,35^

In our study, patients experiencing fragmented care had improved overall survival compared with those with fully coordinated care. This survival benefit may be explained by higher rates of adjuvant chemotherapy among fragmented care patients, as receiving treatment closer to home after surgery at a HVH likely reduced travel burden and improved access to timely therapy. In contrast to prior assumptions, this study suggests that care fragmentation may facilitate access to adjuvant therapy in some circumstances. Care fragmentation was associated with increased receipt of adjuvant chemotherapy and improved survival for patients with PDAC treated at HVHs. This may reflect the ability to initiate systemic therapy locally after surgery at a HVH, reducing travel burden and allowing for more convenient treatment for patients.^32^ These findings suggest that, in some cases, fragmentation of care may overcome barriers to receiving multi-modal treatment, a conclusion that contrasts previous literature describing care fragmentation as a “crisis” in the United States healthcare system.^36,37^ While fragmentation can compromise care when it results from poor communication or lack of coordination, it may also represent a pragmatic solution in settings where centralized surgery is necessary, but ongoing treatment closer to home is preferable or more feasible. Advances in health information technology and the widespread use of electronic medical records and patient portals may help enable this model by supporting more effective care transitions across facilities.^38^ By improving access to medical records and communication across institutions, these tools can help patients receive necessary postoperative therapy closer to home, reducing both financial strain and travel-related challenges.^39^ Moreover, prior studies have demonstrated that the location of adjuvant chemotherapy,whether at the index surgical hospital or a local facility,is less important than ensuring the therapy is received.^36^ Future research should evaluate which models of post-surgical care coordination most effectively balance centralization with access to local systemic therapy.

Despite national guideline efforts, profound variation in the receipt of adjuvant therapy for pancreatic cancer persist. This study demonstrated that patients treated at HVH who are uninsured, enrolled in Medicaid, belong to lower income brackets, or identify as non-Hispanic Black were significantly less likely to receive adjuvant chemotherapy. These findings align with previous research demonstrating that socioeconomic and racial barriers hinder access to guideline-concordant pancreatic cancer care and that receiving care at an HVH does not eradicate these associations.^40^ Unfortunately, such variation in treatment utilization is not unique to pancreatic cancer and has been documented across a range of malignancies in the United States.^41,42^ The root cause of this variation is complex and multifactorial and may include disparities in referral patterns to medical oncologists, diminished patient–provider trust, and greater travel distances required for patients from marginalized communities to access specialized cancer care. Despite these challenges, the increase in adjuvant chemotherapy use for PDAC, from 59% in 2006 to 69% in 2017, signals growing adherence to national cancer guidelines and is similar to trends reported in other studies.^43^ Nonetheless, significant gaps persist, particularly among socioeconomically disadvantaged patients and those facing logistical barriers to comprehensive care.

While findings regarding care fragmentation in this study are striking, it is important to note that this is in a specific use case of patients undergoing upfront pancreatectomy for PDAC. This test case was selected because it represents a well-defined cohort with standardized treatment pathways, enabling clearer attribution of outcomes to care delivery patterns. Patients receiving upfront resection offer a uniform entry point into the healthcare system, minimizing confounding related to neoadjuvant therapy. However, as the use of neoadjuvant therapy continues to expand in resectable PDAC, the dynamics of fragmentation may shift, since systemic therapy would already be initiated prior to surgery and local chemotherapy administration may play a different role. Future work should evaluate whether the benefits observed here extend to neoadjuvant-treated cohorts. However, creative study designs that incorporate patients receiving neoadjuvant therapy, or managed at lower volume hospitals, may further define the generalizability of multimodal care decentralization as a strategy to mitigate access disparities. Future studies should explore whether similar benefits of decentralized care persist in other oncologic contexts, where care pathways are less standardized and more dependent on multidisciplinary coordination.

This study has important limitations. First, it is a retrospective analysis of the NCDB, which includes only CoC-accredited institutions and lacks certain clinical details such as disease recurrence and cancer-specific survival. Second, travel distance was estimated using ZIP code centroids, limiting geographic precision and may underestimate travel burden. Third, while the NCDB captures whether adjuvant chemotherapy was initiated, it does not include information on specific regimen, duration, or treatment completion, which limits our ability to assess treatment adherence or intensity. Fourth, the fragmented care variable is limited since it does not capture hospital-specific information on the secondary institution at which a patient received treatment, and the assumption that modalities of care were spread across multiple reporting facilities is not directly measurable in the dataset.

## Conclusion

In patients undergoing upfront resection of PDAC at HVHs, sociodemographic characteristics and longer travel distances were associated with poorer administration of systemic therapy and overall survival. Fragmentation of care was associated with improved rates of adjuvant chemotherapy delivery and overall survival. As complex surgical care continues to be centralized, necessitating longer travel burdens for patients, thoughtful and well-coordinated care fragmentation may improve access to and delivery of multimodality care.

## Supplementary Information

Below is the link to the electronic supplementary material.

Supplementary file1 (DOCX 233 kb)
