# Supplementary material for: Association Between Fragmentation of Care and Delivery of Adjuvant Chemotherapy in Patients Traveling to High-Volume Hospitals for Pancreatic Adenocarcinoma
Source: Ann Surg Oncol. 2025 Oct 16;33(2):1576–85. doi: 10.1245/s10434-025-18539-4 (PMC12765744; doi:10.1245/s10434-025-18539-4)
Supplement: Supplementary file 1 — Supplementary file1 (DOCX 233 kb) [file 10434_2025_18539_MOESM1_ESM.docx]

**Supplemental Figure 1**: Nonlinear Association Between Travel Distance and Mortality Risk: LOWESS vs Linear Fit


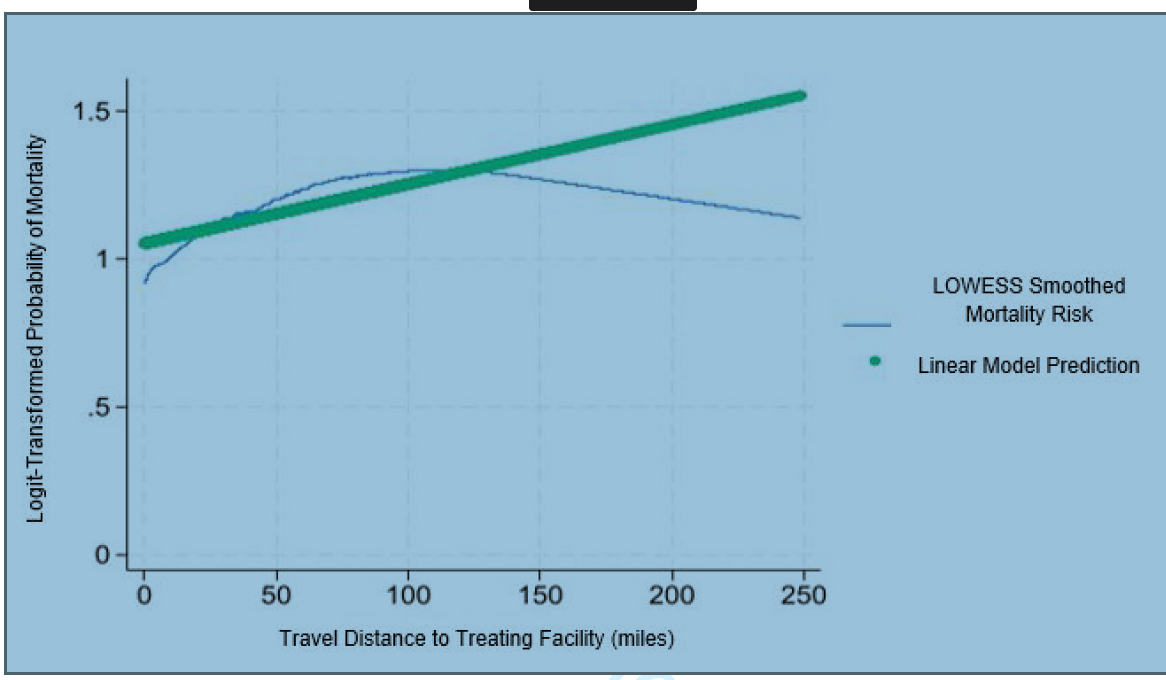


The Lowess smoothed line (blue line) in the graph deviates from a straight-line trend (green line), indicating a nonlinear relationship between travel distance and mortality probability. Initially, mortality probability increases at shorter distances, then plateaus, and eventually declines at greater distances—a pattern that a simple linear model (green points) fails to capture. Since this nonlinearity suggests different effects at different distance ranges, stratifying distance into deciles helps analyze mortality risk more accurately by comparing trends within distinct distance groups rather than assuming a uniform effect.
